# A Hyperspectral Imaging Approach for Classifying Geographical Origins of Rhizoma Atractylodis Macrocephalae Using the Fusion of Spectrum-Image in VNIR and SWIR Ranges (VNIR-SWIR-FuSI)

**DOI:** 10.3390/s19092045

**Published:** 2019-05-01

**Authors:** Chenlei Ru, Zhenhao Li, Renzhong Tang

**Affiliations:** 1 Department of Industrial and Systems Engineering, Zhejiang University, Hangzhou 310058, China; tangrz@zju.edu.cn; 2 Pharmaceutical Informatics Institute, College of Pharmaceutical Sciences, Zhejiang University, Hangzhou 310058, China; zhenhao@zju.edu.cn

**Keywords:** hyperspectral imaging, data fusion, traditional Chinese medicine, Rhizoma Atractylodis Macrocephalae, geographical origin classification

## Abstract

Hyperspectral data processing technique has gained increasing interests in the field of chemical and biomedical analysis. However, appropriate approaches to fusing features of hyperspectral data-cube are still lacking. In this paper, a new data fusion approach was proposed and applied to discriminate Rhizoma Atractylodis Macrocephalae (RAM) slices from different geographical origins using hyperspectral imaging. Spectral and image features were extracted from hyperspectral data in visible and near-infrared (VNIR, 435–1042 nm) and short-wave infrared (SWIR, 898–1751 nm) ranges, respectively. Effective wavelengths were extracted from pre-processed spectral data by successive projection algorithm (SPA). Meanwhile, gray-level co-occurrence matrix (GLCM) and gray-level run-length matrix (GLRLM) were employed to extract textural variables. The fusion of spectrum-image in VNIR and SWIR ranges (VNIR-SWIR-FuSI) was implemented to integrate those features on three fusion dimensions, i.e., VNIR and SWIR fusion, spectrum and image fusion, and all data fusion. Based on data fusion, partial least squares-discriminant analysis (PLS-DA) and support vector machine (SVM) were utilized to establish calibration models. The results demonstrated that VNIR-SWIR-FuSI could achieve the best accuracies on both full bands (97.3%) and SPA bands (93.2%). In particular, VNIR-SWIR-FuSI on SPA bands achieved a classification accuracy of 93.2% with only 23 bands, which was significantly better than those based on spectra (80.9%) or images (79.7%). Thus it is more rapid and possible for industry applications. The current study demonstrated that hyperspectral imaging technique with data fusion holds the potential for rapid and nondestructive sorting of traditional Chinese medicines (TCMs).

## 1. Introduction

Hyperspectral imaging (HSI) is an analytical tool merging conventional imaging and spectroscopy to simultaneously provide physical features and internal chemical composition of the detected object [1]. Conventional multispectral imaging, such as RGB imaging, obtains information in certain distinct wavelengths. In contrast, HSI collects data over a continuous spectral range in hundreds of wavelengths. The hyperspectral data-cube has a shape X × Y × λ, with X (width) and Y (height) representing two coordinates of spatial images and λ representing spectral wavelengths [2]. Although HSI provides both spectral and image information, most classification applications use only the former. Recently, several discrimination methods based on spectrum and image integration have been reported, such as land cover analysis in remote sensing [3,4], variety distinction of rice [5] and corn seed [6], geographical origin identification of *Jatropha curcas L.* seeds [7]. However, these studies focus on HSI systems within the visible and near-infrared (VNIR, about 400–1000 nm) range, or the short-wave near-infrared (SWIR, about 900–1700 nm) range. Moreover, there have been few HSI studies on comparing the classification performance of spectrum and image fusion with VNIR and SWIR range combined, especially in the realm of traditional Chinese medicine (TCM) due to lack of appropriate methodology [8,9,10].

Rhizoma Atractylodis Macrocephalae (RAM) is one of the most commonly used herbal medicines in China. It has shown various pharmacological activities including immunomodulatory [11], anti-oxidant [12], anti-inflammatory [13], and gastro-protective [14] effects. It is worth noting that the contents of bioactive compounds of RAM are affected by geographical origins [15,16,17], which may impair therapeutic consistency. Therefore, identification of geographical origins is highly critical to ensure quality and clinical efficacy of RAM. RAM is mainly cultivated in Zhejiang, Anhui, and Hebei Province of China [18], and those produced in Zhejiang are considered to be of better quality. As RAM slices from different origins can be mingled in commodity circulation, an effective and reliable approach is urgently needed to trace the origins.

Traditionally, the origins of TCMs are identified based on physical characteristics in macroscopical and microcosmic morphology, as well as color, odor, taste, weight, and density. These methods are direct, but subjective and inaccurate. Modern analytical methods including high-performance liquid chromatography (HPLC) [19], gas chromatography-mass spectrometry (GC-MS) [20], and liquid chromatography-mass spectrometry (LC-MS) [21], have also been increasingly employed for origin identification using marker compounds quantification and/or fingerprinting analysis. Although these methods are reliable and accurate, the analytical cost is high, and the analytical procedure is generally labor-intensive with inevitable sample consumption. More recently, several rapid spectroscopic technologies, such as near-infrared spectroscopy (NIRS), have been gradually introduced into the classification of TCM origins [22,23]. However, most of these methods still need powdered samples for detection, which leads to the loss of external spatial information, as well as sample destruction. 

HSI system in this study employs both VNIR and SWIR ranges. Hyperspectral data-cube in each range contains both spectral and image information. Therefore, it is feasible to integrate data from spectral and image in different ranges, thus improving the classification performance while reducing the environmental sensitivity (temperature, humidity) of spectroscopy to some extent.

The main purpose of this paper was to develop an effective and reliable HSI method to classify geographical origins of RAM slices. Firstly, hyperspectral data of the samples were acquired in both VNIR (435–1042 nm) and SWIR (898–1751 nm) ranges. The regions of interest (ROIs) in both ranges were identified respectively. Then, the spectral features corresponding to ROIs were pre-processed by five pre-processing algorithms. Next, successive projections algorithm (SPA) was applied to gain effective wavelengths. In the same ROIs, texture features were extracted by gray-level co-occurrence matrix (GLCM) and gray-level run-length matrix (GLRLM). After all features were acquired, the fusion of spectrum-image in VNIR and SWIR ranges (VNIR-SWIR-FuSI) was performed, in three different ways: (1) VNIR and SWIR fusion, (2) spectrum and image fusion, and (3) all data fusion. Finally, Partial least squares-discriminant analysis (PLS-DA) and support vector machine (SVM) were used as classification models. The enhancement of VNIR-SWIR-FuSI was discussed in two parts: full bands, SPA bands. In addition, classification maps were used to visualize different RAM slice origins based on data fusion.

## 2. Materials and Methods

### 2.1. Hyperspectral Imaging System

**Figure 1:**
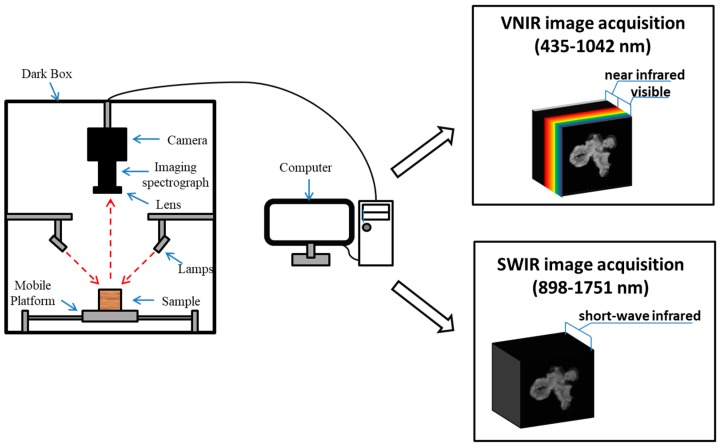
The hyperspectral imaging (HSI) system in reflection mode (VNIR: the visible and short-wave near-infrared, SWIR: long-wave near-infrared).

An HSI system combining VNIR (435–1042 nm) and SWIR (898–1751 nm) spectral ranges in reflection mode was used in the study (Figure 1). The system consisted of an imaging module, two 150W halogen lamps (3900ER, Illumination Technologies Inc., Boxborough, MA, USA), a horizontal mobile platform (ETH14, TOYO, Foshan, China), and a default image acquisition software (Spectral Image, Isuzu Optics, Xinzhu, China). The imaging module comprised a visible spectral camera (Zyla-4.2-Plus, Andor, Abingdon, Oxon, UK), an adjustable visible focal lens (OLE23, Schneider, Luemerson, Paris, France), a near-infrared spectral camera (OWL-640-mini, Raptor, Belfast, Northern Ireland, UK), an adjustable near-infrared focal lens (OLES23, Specim, Oulu, Finland), and an imaging spectrograph (ImSpector-V10E, Specim, Oulu, Finland). 

The operation parameters in the VNIR range were set as follows: speed of the mobile platform 13 mm/s, distance between the lens and the sample 25 cm, exposure time 17 ms. Hyperspectral data-cubes were obtained with 512 pixels wide, 1200 pixels long, and 237 wavelengths ranging from 435 to 1042 nm at intervals of 2.60 nm. The pixel size was 0.16 mm × 0.16 mm. The parameters in the SWIR range were set as follows: speed of the mobile platform 4.5 mm/s, distance between the lens and the sample 25 cm, exposure time 42 ms. Hyperspectral data-cubes were obtained with 640 pixels wide, 1500 pixels long, and 512 wavelengths ranging from 898 nm to 1751 nm at intervals of 1.67 nm. The pixel size was 0.23 mm × 0.23 mm. In fact, for either of two ranges, the length of the distance that mobile platform moved varied among samples even if the start and end point were fixed. Therefore, the lengths of data-cubes were padding to 1200 and 1500 for VNIR and SWIR ranges, respectively, for consistency. 

### 2.2. Sample Preparation

**Figure 2:**
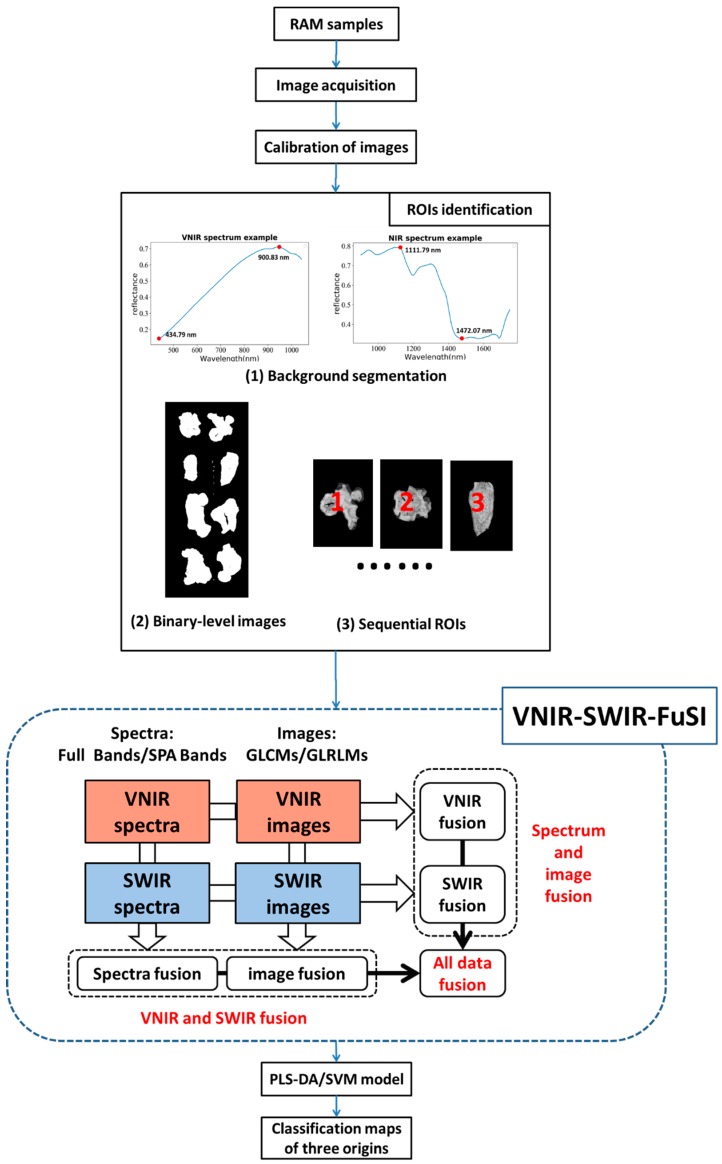
Flow chart of the experimental procedure used to classify different geographical origins of RAM slices. (ROI: region of interest, SPA: successive projections algorithm, GLCM: gray-level co-occurrence matrix, GLRLM: gray-level run-length matrix analysis, PLS-DA: partial least square-discriminant analysis, SVM: support vector machine).

Rhizoma Atractylodis macrocephalaes (the rhizoma of *Atractylodes macrocephala* Koidz, also known as white atractylodes) from Anhui, of China were provided by Jiuzhou Fangyuan (Hefei, China). All the RAMs were fully washed, cut into slices and sun-dried. The samples were placed in plastic bags and stored at room temperature before use. There were 16 batches, six batches and six batches from Anhui, Zhejiang, and Hebei respectively. A total of 224 RAM slices (8 RAM slices for every batch) were randomly picked. Samples from each geographical origin were randomly divided into a training set and testing set by a ratio of 2:1. Finally, 149 RAM slices and 75 RAM slices were used for calibration and prediction, respectively. Figure 2 presents the flow chart of the experimental procedure.

### 2.3. Image Preprocessing

#### 2.3.1. Image Calibration

The acquired reflectance images were affected by an illumination source, the dark current of the charge coupled device (CCD) cameras, as well as the differences in the physical configuration of the hyperspectral imaging system. Therefore, the images were calibrated with the white and dark reference images as follows:

(1) $$R_{c}=\frac{R_{\mathrm{raw}}{-R}_{\mathrm{dark}}}{R_{\mathrm{white}}{-R}_{\mathrm{dark}}}$$

where R_c_ is the calibrated reflectance image, R_raw_ is the raw reflectance image, R_white_ is the white reference image, and R_dark_ is the dark reference image. The white reference image was acquired using a Teflon whiteboard with nearly 100% reflectivity. The dark reference image was acquired by covering the lens with cap. 

#### 2.3.2. ROIs Identification

After the calibration of images, the ROI of each RAM slice was identified automatically in Spyder (Python 3.6). In this study, the processes of ROI identification included background segmentation, transferring to binary-level images, and sequential ROI extraction of each RAM slice. In order to maximize the contrast of relative reflectance intensity between background and samples, new gray-level images were generated by subtracting the images with lowest intensity from those with highest intensity. Then binary threshold segmentation was applied to new gray-level images to remove background and gain binary-level images. Corresponding ROIs of RAM slices in binary-level images were extracted by the built-in function “findContours” of Spyder (Python 3.6).

### 2.4. Extraction of Spectral Features

In this study, two steps were used for the extraction of spectral features. Firstly, spectra were pre-processed by five pre-processing methods. Then effective wavelengths were selected by SPA.

#### 2.4.1. Spectral Pre-Processing

Spectra in 898–1042 nm of the VNIR range and in 1601–1751 nm of the SWIR range were not included due to low signal-to-noise ratios. Besides, a few pixel values of gray-level images at 898 nm of the SWIR range were nonnumeric. Thus, the reserved spectral ranges were 435–898 nm with 182 wavelength bands, 900–1601 nm with 421 wavelength bands for VNIR and SWIR, respectively. 

Due to the noises from electromagnetic radiation of cameras and uneven surface of samples, spectra in VNIR and SWIR ranges were significantly influenced by scatter effects which led to baseline shift and non-linearity [24]. Suitable pre-processing methods can largely eliminate such effects, thus improving the subsequent classification models. The most common pre-processing methods can be divided into two categories: scatter-correction and derivatives. Scatter-correction methods include smoothing [25], standard normal variate transformation (SNV) [26], and multiplicative scatter correction (MSC) [27]. Derivatives methods include first derivative [19] and second derivative [28]. All the pre-processing technologies mentioned above were evaluated in this study. Smoothing methods were Savitzky-Golay (SG) smoothing with polynomial order three of 9-point, 13-point, 17-point, and 21-point respectively. And the best smoothing filter (17-point) was also implemented before the first and second derivative operation. 

#### 2.4.2. Effective Wavelength Selection

In order to reduce computation load and eliminate redundant information of hyperspectral data, effective wavelengths were chosen. SPA has been regarded as a powerful waveband selection method, which can minimize the multi-collinearity among variables [29]. Therefore, SPA was used herein for optimal wavelength selection to improve prediction accuracy and calculation speed of classification models. This procedure was carried out in MATLAB (The Mathworks, Inc., Natick, MA, USA). 

### 2.5. Extraction of Image Features

Textural features extracted by GLCM and GLRLM were employed for geographical origin identification. GLCM is a classic method for extracting textural properties, which can describe intensity change in the local spatial domain [30]. GLCM textures include the contrast, dissimilarity, homogeneity, energy, and correlation extracted from four directions (0°, 45°, 90°, 135°) by Spyder (Python 3.6). Twenty parameters could be obtained from GLCM. GLRLM is another texture analysis technique, which provides several state-of-the-art high-order statistics [31,32]. In this approach, the information on the run of the same gray-level value in a specific direction is contained in a GLRLM. The length of the run is the number of pixels in the run. Coarse texture features are dominated by long runs, while fine texture features are populated by short runs. From GLRLM, a set of seven scalar texture measures was computed, which includes short run emphasis, long run emphasis, gray-level non-uniformity, run percentage, run length non-uniformity, low gray level run emphasis and high gray level run emphasis.

In most cases, GLCM and GLRLM are used for extracting textural features from mono-spectral images. However, hyperspectral images have hundreds of bands. If GLCM and GLRLM textures are calculated for all gray-level images corresponding to their bands, there will be a mass of redundant information, which will increase the computing complexity. Therefore, only texture information in the gray-level image in each effective wavelength was extracted in this study. 

### 2.6. The Fusion of Spectrum-Image in VNIR and SWIR Ranges (VNIR-SWIR-FuSI)

Data fusion has gained rising interest due to the boost it gives to multiple analysis tasks. The fusion of data from various sources can provide complementary information and increase the robustness and accuracy of the built models [33]. In this study, a novel method called VNIR-SWIR-FuSI was developed to fuse hyperspectral data in three dimensions: VNIR and SWIR fusion, spectrum and image fusion, and all data fusion. VNIR and SWIR fusion was implemented to improve classification performance by combining spectral and image features in the VNIR or SWIR range only. Spectrum and image fusion was used for promoting classification models through integrating spectral and image features in both VNIR and SWIR ranges. All data fusion referred to the integration of spectra and images in both VNIR and SWIR ranges. Besides, the results of hyperspectral data in effective wavelengths using VNIR-SWIR-FuSI were also compared with those in full wavelengths. The VNIR-SWIR-FuSI approach had a clear promotion in the classification performance in both all wavelengths and effective wavelengths, and the enhancement of the latter was more significant than the former. 

### 2.7. Classification Models

In this work, supervised pattern recognition models were adopted for the origin identification of RAM slices. There have been a variety of models available for classification, including partial least square-discriminant analysis (PLS-DA) [34,35], linear discriminate analysis (LDA) [36], support vector machine (SVM) [8] and back propagation neural network (BPNN) [5]. PLS-DA and SVM were selected herein. 

PLS-DA is an adaptation of PLS regression to the problem of supervised classification. PLS-DA is performed in order to sharpen boundaries among classes of observations by projecting the input features to the most discriminative directions [37]. SVM is a classical machine learning method used for classification, regression and outlier detection. The main processes of SVM include mapping input vector to a high-dimensional feature space and then using an optimal hyper-plane to perform separation [38]. For PLS-DA, the number of latent variables was chosen from all integers under 10. For SVM with RBF kernel function, the penalty parameter C and kernel coefficient g were both set to a series of discrete values in the interval from 0.001 to 10. Auto-scaling (unit variance scaling combined with mean centering) [33] was selected as the default data processing method for both classification models to eliminate the influence of variable dimension. All algorithms were implemented using programs developed in Spyder (Python 3.6). In order to generalize the model, ten-time five-fold cross-validation was applied. 

### 2.8. Evaluation of Classification Models

**Figure 3:**
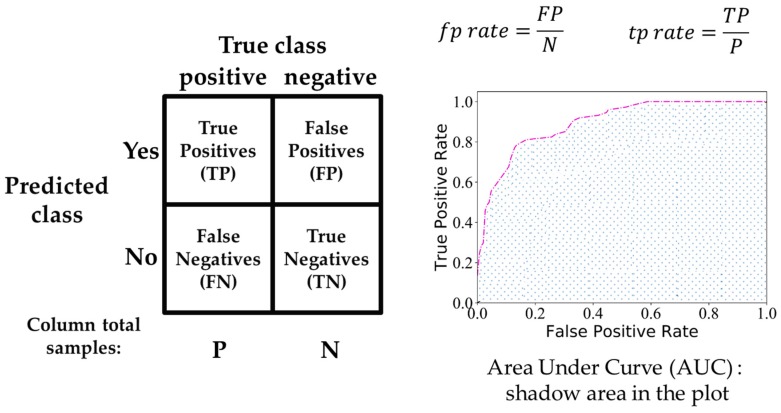
Confusion matrix (**left**) and a sketch of ROC curves (**right**).

Classification accuracy is widely used to evaluate model performance, while it has some limitations in practical applications, especially for class distribution imbalance problem and unequal classification error costs [39]. Therefore, in addition to classification accuracy, this work employed a receiver operating characteristics (ROC) [40] as an extension of metrics to visualize and select classification models based on their performance. ROC curves are two-dimensional graphs, which plot true positives rate (tp rate) on the vertical Y-axis against false positives rate (fp rate) on the horizontal X-axis. The area under the curve (AUC) is the shadow area shown in Figure 3. The performance of classifiers was positively correlated with the value of AUC. 

### 2.9. Visualization of RAM Geographical Origins

It is obvious that the geographical origins of RAM slices are difficult to be identified by the naked eye. Classification map [26] can be used to visualize each pixel of hyperspectral images to recognize different origins, which is considered to be superior to conventional spectroscopy methods. In the present work, hyperspectral images were reconstructed for visualization by compressing pixel blocks to pixel points. In order to get the same resolution for VNIR and SWIR ranges, the value of new pixel points was set to the average of 4 × 4 and 5 × 5 pixel blocks respectively. Then the PLS-DA calibration models on full bands were used to produce classification maps in these two ranges. The classification maps were displayed in three primary colors (red representing Anhui, green representing Zhejiang, and blue representing Hebei). In this way, people can easily differentiate the origins of RAM slices by the color variation in the generated maps. All steps involved were implemented using programs developed in Spyder (Python 3.6). 

## 3. Results and Discussion

### 3.1. Representative RGB Images and Raw Spectra of RAMs

**Figure 4:**
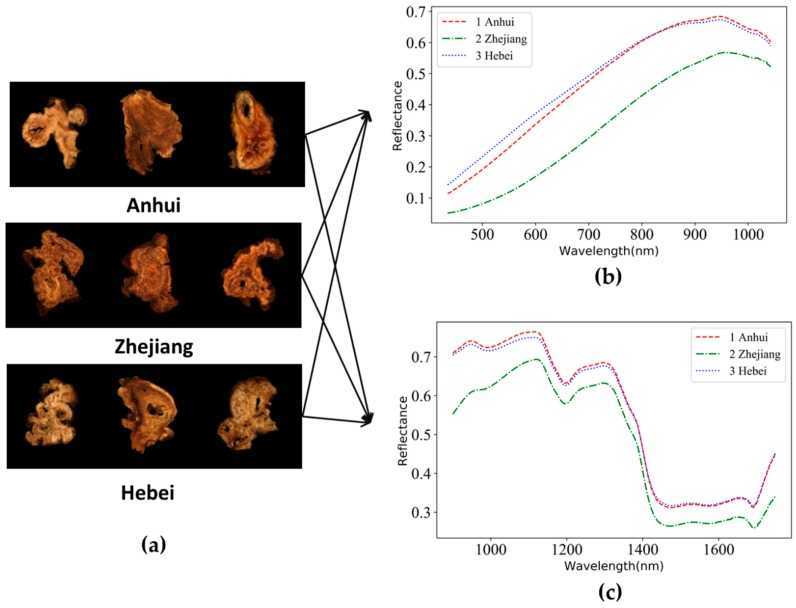
Representative Red-Green-Blue (RGB) images (**a**) and raw spectra of Rhizoma Atractylodis Macrocephalaes (RAMs) in the VNIR range (**b**) and SWIR range (**c**) based on the whole data set.

The representative RGB images of RAMs from three different geographical origins are shown in Figure 4a. RAMs from different origins can hardly be distinguished by their appearance characteristics. The comparison results of the three spectral curves between three origins show certain differences. For example, the RAMs from Zhejiang exhibited lowest reflection intensities both in VNIR and SWIR ranges, which significantly varied from those of Anhui and Hebei. Spectral curves corresponding to Anhui and Hebei had similar trends. Spectral curves of Anhui and Hebei can be separated at wavelengths from 435 nm to 747 nm in the VNIR range, but nearly overlapped in the range of 747–898 nm (Figure 4b). Moreover, these two curves can be separated in the range of 900–1130 nm and 1225–1310 nm, but with little difference in the remaining spectral range (Figure 4c). The spectral diversity in VNIR and SWIR ranges was possibly due to the difference in contents of phytochemicals (atractylenolide I, II, III [41], atractylon [42], polysaccharide [43] and so on) and physical factors (e.g., uniformity, density) [6,38] of the RAM samples. 

### 3.2. Selection of Pre-Processing Methods

Pre-processing of spectral data is a crucial step prior to chemometrics modeling. Table 1 shows the prediction accuracies corresponding to pairwise combinations of five pre-processing algorithms (SNV, MSC, SG smoothing, first derivative, and second derivative) and classification models including PLS-DA and SVM, which were calculated in both VNIR and SWIR ranges.

As shown in Table 1, the overall performance of spectra pre-processed by derivatives was better than that of other methods. In more detail, the result of second derivative was slightly better than the result of first derivative. Therefore, second derivative was selected as the pre-processing method.

### 3.3. Wavelength Selection

In order to minimize redundant information of hyperspectral data, effective wavelengths were selected by SPA. This approach has been used as an effective variable selection algorithm to solve the collinearity problem of hyperspectral data. The selection of effective wavebands is shown in Figure 4. 

**Figure 5:**
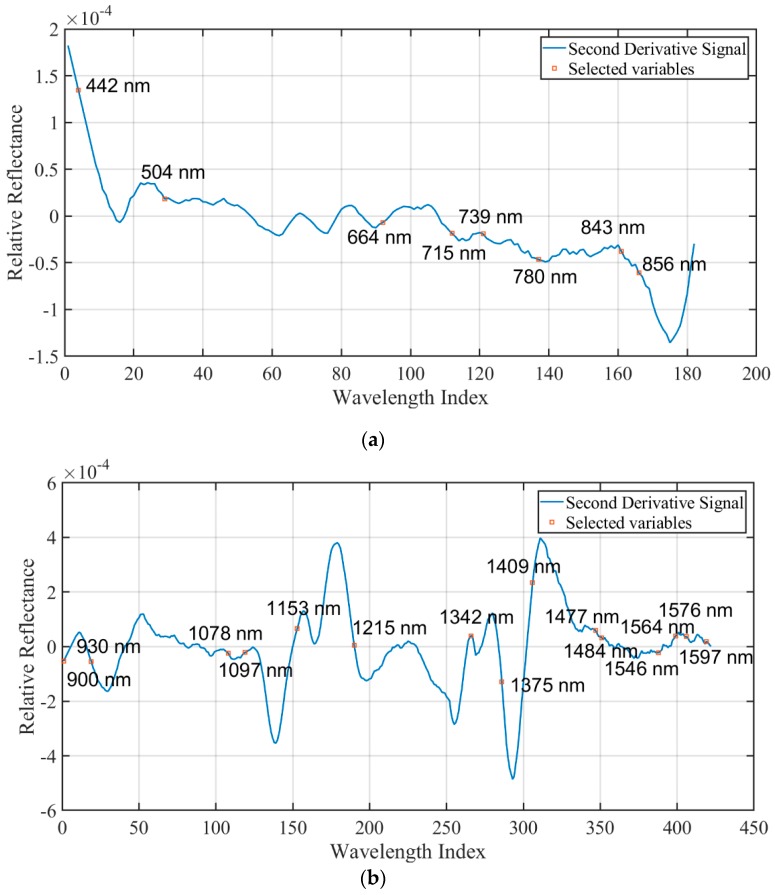
Effective wavelengths extracted from the VNIR range (435–898 nm) (**a**) and the SWIR range (900–1601 nm) (**b**) using successive projection algorithm (SPA) based on the calibration set.

The interpretation of selected wavelengths referred to the work of Workman et al. [44]. Figure 5a shows that there were eight effective wavelengths (442, 504, 664, 715, 739, 780, 843, 856 nm) obtained in the VNIR range (435–898 nm). The wavelengths at 442, 504, 664, 715 and 739 nm demonstrated that geographical origins of RAM slices can be to some extent differentiated in visible range. Wavelengths at 780 nm corresponded to N-H stretching (third overtone) of amino acids, and wavelengths at 843 and 856 nm corresponded to absorption of ArC-H (third overtone) in resins as well as absorption of methyl (third overtone). Figure 5b shows that there were fifteen effective wavelengths (900, 930, 1078, 1097, 1153, 1215, 1342, 1375, 1409, 1477, 1484, 1546, 1564, 1576, 1597 nm) obtained in the SWIR range (900–1601 nm). The wavelengths at 900, 930 nm corresponded to C-H stretching (third overtone), O-H stretching (third overtone) of polysaccharides and resins. The wavelengths at 1078, 1097, 1153 and 1215 nm corresponded to C-H stretching (second overtone). The wavelengths at 1342, 1375 and 1409 nm corresponded to C-H stretching (first overtone combinations), O-H stretching (1st overtone) of H_2_O, polysaccharides, and resins. The wavelengths at 1477, 1484, 1546 and 1564 nm corresponded to N-H stretching (first overtone) of amino acids and O-H stretching (first overtone) of polysaccharides. The wavelengths at 1576, 1597 nm corresponded to O-H stretching (first overtone) of resins.

### 3.4. Full Bands Based Classification

In this section, VNIR-SWIR-FuSI was used for classification purpose in full bands. The classification performance using VNIR and SWIR fusion, spectrum and image fusion, and all data fusion was compared to that based solely on spectral or textural features, respectively. Table 2 summarized that: (1) VNIR and SWIR fusion showed no effect on classification performance, (2) spectrum and image fusion improved the prediction performance in the VNIR range, and (3) all data fusion got the best correct rate of 97.3%, which was superior to those based on spectra (93.2%) or textures (79.7%) only. The results of ROCs are consistent with those of classification accuracies, and all data fusion got the best AUC value of 0.995, which was also better than those based on spectra (0.987) or textures (0.941) only.

#### 3.4.1. Classification with VNIR and SWIR Fusion

The effect of VNIR and SWIR fusion was evaluated in two parts: spectra (A), and images (B). Table 2 (A) shows that better performance of PLS-DA can be achieved when the fusion method was adopted. The highest accuracy of PLS-DA was 94.6%. However, spectral range fusion failed to improve SVM performance, the accuracy of which (92.4%) was slightly lower than that in the SWIR range only. Table 2 (B) shows that the fusion method seemed useless on neither GLCM textures nor GLRLM textures for both models. Both Table 2 (A) and Table 2 (B) shows that the fusion method could not improve the discrimination result. A probable explanation was that spectral and textural features under both VNIR and SWIR ranges were similar and could not work complementarily to improve classification performance. 

#### 3.4.2. Classification with Spectrum and Image Fusion

The texture features were acquired from hyperspectral images using GLCM and GLRLM. The fusion effects of spectra in tandem with GLCM or GLRLM textures were evaluated separately in two parts: VNIR (I), and SWIR (II). For the GLCMs in Table 2 (I), the fusion method increased the accuracy of SVM, while Table 2 (II) shows the fusion method did not work. Thus, spectra combined with GLCMs seemed to have limited effects. For the GLRLMs in Table 2 (I), the accuracies corresponding to PLS-DA and SVM were 86.5%, 84.6% before fusion, and 90.5%, 89.7% after fusion respectively. However, there was no significant difference before and after fusion, as shown in Table 2 (II). Thus, spectra combined with GLRLMs could enhance the classification performance in the VNIR range, but not in the SWIR range. 

#### 3.4.3. Classification with All Data Fusion

The all data fusion method integrated spectral and textural variables in both VNIR and SWIR ranges. Fusion effects could be discussed in two parts due to two different series of textural features. The results were presented where Table 2 (III) and Table 2 (C) overlapped. The overlap shows that the fusion method was not effective to improve the results of spectra combined with GLCMs. However, the fusion method improved the results by the integration of spectra and GLRLMs. After the fusion with GLRLMs, the global optimal accuracies of 97.3% and 96.2% were achieved by PLS-DA and SVM respectively. The result shown in Section 3.4.2 and Section 3.4.3 indicate that the texture features extracted by GLRLM were more suitable for data fusion than those extracted by GLCM. 

#### 3.4.4. ROC Curves of Three Fusion Methods

**Figure 6:**
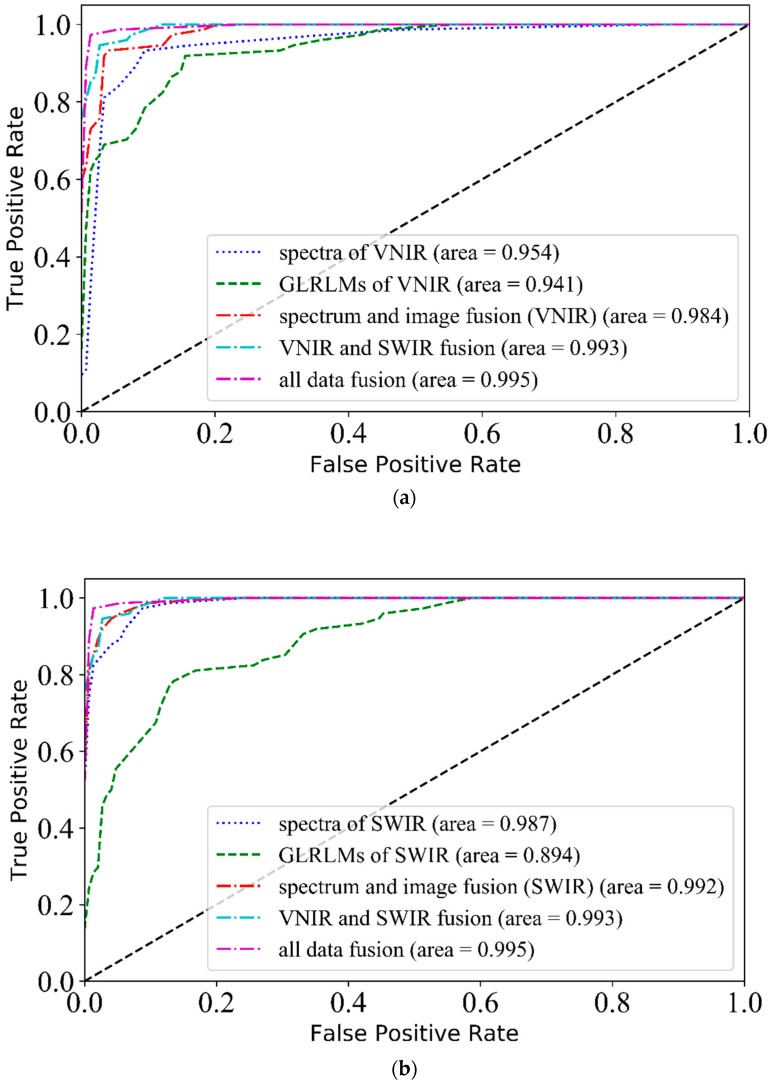
The receiver operating characteristics (ROC) curves of three fusion methods, full band spectra, and images in (**a**) the VNIR range and (**b**) the SWIR range based on the prediction set.

Besides classification accuracies, ROC curves were used to evaluate the fusion methods. ROC curves were plotted based on PLS-DA classifier due to its better performance, simple calculation and highly interpretability. As GLRLMs were turned out to be superior to GLCMs above, the image features referred in particular to GLRLMs only. Figure 6 shows that the three fusion methods achieved better AUCs than those of spectra or images in one spectral range, and all data fusion method obtained the optimal AUC value of 0.995. The conclusion of ROC curves was consistent with that of classification accuracies with one difference: classification accuracies showed that all data fusion method was effective to the SWIR range, but the other two fusion methods made no difference, while ROC analysis indicated that all the three fusion methods were effective. One explanation was that ROC curves revealed better performance hidden in the fusion methods. Moreover, by summarizing the results of classification accuracies and ROC curves, the spectral features in the SWIR range were more efficient than those in the VNIR range for classification. In contrast, features in VNIR were more efficient in the GLRLM textures. All data fusion method included the two efficient feature parts, thereby showing superior performance to other fusion methods.

### 3.5. SPA Bands Based Classification

VNIR-SWIR-FuSI was also used to discriminate origins in SPA bands. As shown in Table 3, when only spectral features were used, the classification results of SPA bands were weaker than those of full bands, and these results were consistent with the empirical results of others [9,10]. This could be attributed to the loss of partial information after SPA features extraction. It can be concluded from Table 3 that: (1) VNIR and SWIR fusion could not improve classification performance, (2) spectrum and image fusion in the VNIR range increased the prediction accuracies, and the improvement of GLRLM textures was better than that of CLCM textures, (3) all data fusion got the best accuracy of 93.2%, and the best AUC value of 0.980 which shows a similar trend to those of full bands. However, SPA used only 23 bands far less than 422 bands of the latter, thus reducing the computation load and eliminating redundant information. 

#### 3.5.1. Classification with VNIR and SWIR Fusion

This section was divided into two parts: spectra (A), images (B). Table 3 (A) shows that fusion methods could always achieve better performance, with a maximum 7.5% increase. However, ROC analysis revealed that VNIR and SWIR fusion had no benefit to the classification performance of spectra. Table 3 (B) was identical to Table 2 (B), which shows VNIR and SWIR fusion could not benefit images on SPA bands. 

#### 3.5.2. Classification with Spectrum and Image Fusion

As shown in Table 3 (I) and (II), spectra in tandem with image features always improved classification accuracies, regardless of GLCMs or GLRLMs. However, the results based on GLRLM were superior to those of GLCM. 

An overview of Table 2 and Table 3 indicates that classification based on image features fared much worse than spectral features. This can be attributed to two possible reasons. The first one is that the appearance of RAM slices from different origins has high similarities [45,46]. The second one could be that the resolution of CCD cameras was not enough to extract more image features. To fetch up these shortages, some state-of-art image processing algorithms can be introduced in further study. For example, convolutional neural network (CNN) [47,48] is a branch of deep learning algorithms in image processing field, which can take original images as input and learn the abstract features automatically. Moreover, CNN can improve the accuracy with the increase of image input. Besides, as an unsupervised learning method, variational auto-encoder (VAE) [49] has a better representation of features with adequate data available, which are more efficient compared with its counterparts, such as GLCM and GLRLM. 

#### 3.5.3. Classification with All Data Fusion

As shown in Table 3, the integration of spectra with either GLCM or GLRLM textures could improve performance. However, the details of GLCM and GLRLM were different. For GLCM, the enhancement of results had little to do with the addition of image features, but mainly due to the spectral fusion as depicted in Section 3.5.1. For GLRLM, the spectral and image features were of equal importance. The highest accuracy of all data fusion was 4.8% better than that of simple spectra fusion. In this section, the same conclusion could be drawn that GLRLMs were of better quality compared to GLCMs. 

#### 3.5.4. ROC Curves of Three Fusion Methods

**Figure 7:**
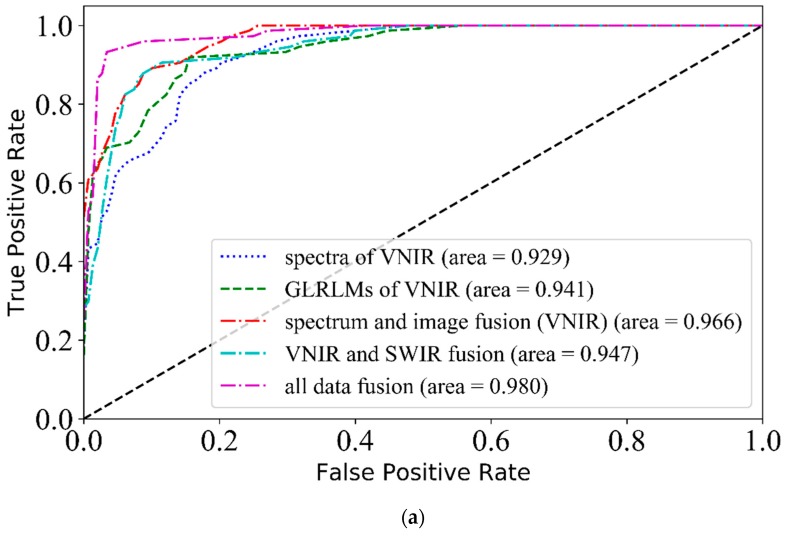

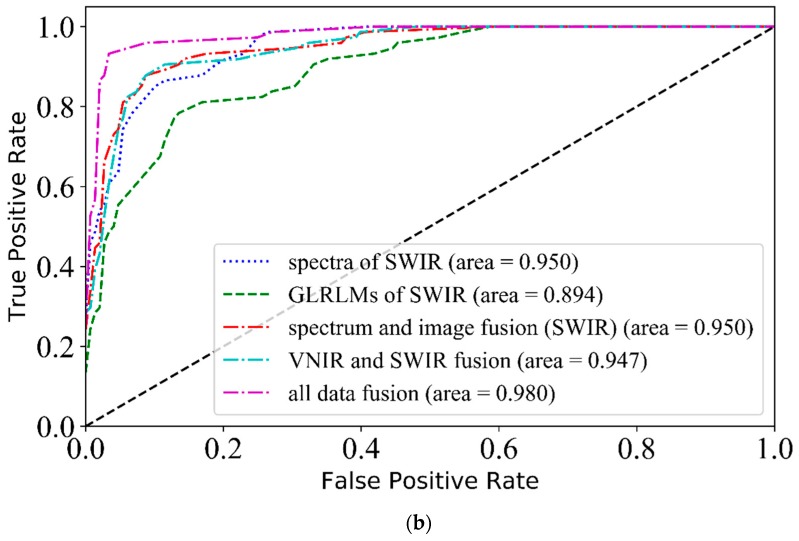
The ROC curves of three fusion methods, SPA band spectra, and images in the VNIR range (**a**) and the SWIR range (**b**) based on the prediction set.

The classifier and image features chosen by ROC curves were the same as Section 3.4.4. Classification accuracies suggested that all three fusion methods improved the performance. However, the results of ROC curves (Figure 7) were not entirely the same as those of classification accuracies. For VNIR and SWIR fusion, the AUC value of the fusion method seemed to take an average between those of spectra in VNIR and SWIR ranges. Thus this fusion method was of little use for SPA bands. Spectrum and image fusion could increase the AUC value of the VNIR range, but not in the SWIR range. All data fusion method showed the best performance, which was similar to the classification accuracy result. It could be seen that spectrum and image fusion in the VNIR range, and all data fusion worked well using either classification accuracies or ROC analysis.

### 3.6. Visualization of RAM Geographical Origins

**Figure 8:**
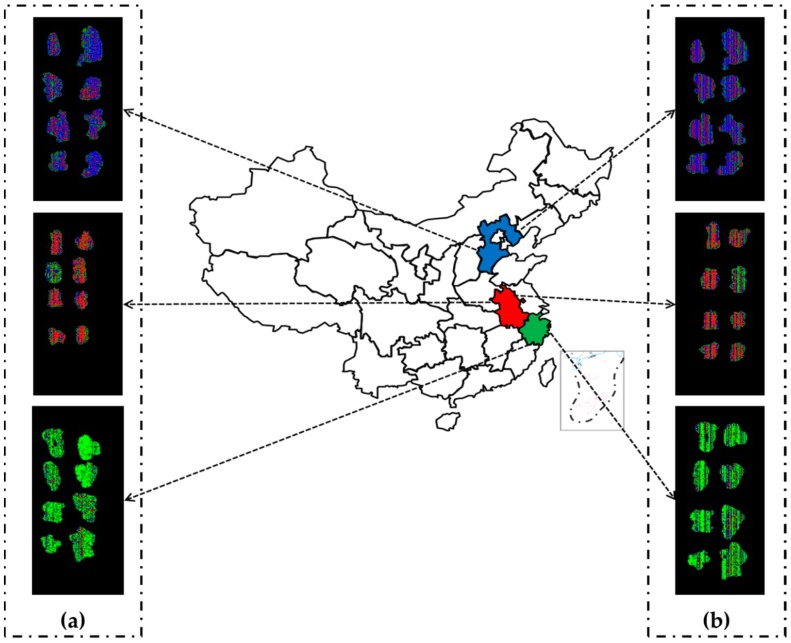
Classification maps by PLS-DA models in the VNIR range (435–898 nm) (**a**) and the SWIR range (900–1601 nm) (**b**) based on the prediction set. The red, green and blue represent Anhui, Zhejiang, and Hebei, respectively. The non-uniform distribution resulted from the categories of pixels not being completely consistent.

Figure 8 visualizes the difference of RAM slices from different geographical origins based on the HSI data, which were marked in three primary colors. It can be observed that RAM slices were represented by a mixture of different colors and had a non-uniform distribution. The result indicates that the HSI imaging system could accurately distinguish RAM slices from different origins in a rapid and nondestructive manner. 

## 4. Conclusions

The geographical origins of RAM slices were classified by the HSI system using VNIR-SWIR-FuSI approach. The results show that data fusion on three dimensions of VNIR-SWIR-FuSI generally had a positive effect on classification performance. The highest classification accuracy of 97.30% was achieved by the PLS-DA model using all data fusion, which was better than those using spectra (93.2%) or textures (79.7%) in one spectral range solely. Although the classification performance of spectra selected by SPA was not so satisfactory (highest 80.9%), the result in SPA bands employing VNIR-SWIR-FuSI could still be fairly good, with highest accuracy of 93.2%. ROC curves also illustrated the reliability of VNIR-SWIR-FuSI in which all data fusion had the greatest AUC values. The optimal result of SPA was almost the same as the result of SWIR spectra. However, as SPA selected only 23 bands which were much less than 422 bands of SWIR spectra, it is possible to develop an online and real-time multi-spectral system for TCMs sorting in further studies. 

## Figures and Tables

**Table 1:** The classification accuracies (%) of pairwise combinations of pre-processing algorithms and classification models in both VNIR and SWIR ranges.

Pre-Processing Algorithms	Models	VNIR		SWIR	
		Calibration	Prediction	Calibration	Prediction
SNV	PLS-DA	81.7	82.4	91.3	87.8
	SVM	84.3	77.3	87.7	84.7
MSC	PLS-DA	81.7	83.8	86.8	86.5
	SVM	83.9	78.9	89.1	82.7
SG (9-point)	PLS-DA	78.1	78.4	77.4	81.1
	SVM	75.7	78.5	78.9	78.6
SG (13-point)	PLS-DA	80.1	79.7	75.7	82.4
	SVM	75.4	84.7	77.9	79.9
SG (17-point)	PLS-DA	80.6	79.7	76.7	83.8
	SVM	75.8	84.1	78.9	79.6
SG (21-point)	PLS-DA	79.8	79.7	77.2	81.1
	SVM	75.3	83.6	76.9	79.5
First Derivative	PLS-DA	82.0	83.8	92.9	93.2
	SVM	85.6	83.2	90.4	92.0
Second Derivative	PLS-DA	86.8	86.5	93.5	93.2
	SVM	85.7	84.6	92.3	93.1

**Table 2:** The classification accuracies (%) in full bands with partial least squares-discriminant analysis (PLS-DA) and support vector machine (SVM) models (Cal and Pre are the abbreviations of calibration dataset and prediction dataset).

Spectral Type	Models	(A) Spectra		(B) Images				(C) Spectrum and Image Fusion			
		Full Bands		GLCM		GLRLM		Full Bands +GLCM		Full Bands +GLRLM	
		Cal	Pre	Cal	Pre	Cal	Pre	Cal	Pre	Cal	Pre
(I) VNIR	PLS-DA	86.8	86.5	78.3	77.0	77.3	79.7	84.8	85.1	86.9	90.5
	SVM	85.7	84.6	76.5	70.1	79.9	76.6	85.2	88.8	86.5	89.7
(II) SWIR	PLS-DA	93.5	93.2	75.5	78.4	74.9	74.3	94.5	91.9	96.5	93.2
	SVM	92.3	93.1	68.4	72.3	76.0	75.9	92.9	89.6	94.1	92.4
(III) VNIR and SWIR Fusion	PLS-DA	93.5	94.6	79.0	77.0	78.1	78.4	92.9	91.9	94.6	97.3
	SVM	93.1	92.4	73.6	71.1	81.5	79.5	92.7	88.2	93.8	96.2

**Table 3:** The classification accuracies (%) in SPA bands with PLS-DA and SVM models (Cal and Pre are the abbreviations of calibration dataset and prediction dataset).

Spectral Type	Models	(A) Spectra		(B) Images				(C) Spectrum and Image Fusion			
		SPA Bands		GLCM		GLRLM		SPA Bands +GLCM		SPA Bands +GLRLM	
		Cal	Pre	Cal	Pre	Cal	Pre	Cal	Pre	Cal	Pre
(I) VNIR	PLS-DA	78.1	75.7	78.3	77.0	77.3	79.7	81.9	86.5	84.7	86.5
	SVM	78.7	80.8	76.5	70.1	79.9	76.6	80.1	81.9	83.3	83.1
(II) SWIR	PLS-DA	82.2	79.7	75.5	78.4	74.9	74.3	87.0	86.5	85.4	83.8
	SVM	86.8	80.9	68.4	72.3	76.0	75.9	81.9	82.3	87.5	88.4
(III) VNIR and SWIR Fusion	PLS-DA	86.5	83.8	79.0	77.0	78.1	78.4	89.1	89.2	89.0	93.2
	SVM	92.0	88.4	73.6	71.1	81.5	79.5	82.0	82.2	88.2	89.6
